# Compensatory mechanisms to maintain glenohumeral joint stability in rotator cuff tears of differing severity during activities of daily living: A musculoskeletal model simulation study

**DOI:** 10.1371/journal.pone.0335647

**Published:** 2025-10-31

**Authors:** Shobu Nakashima, Masayuki Kawada, Yasufumi Takeshita, Takasuke Miyazaki, Masafumi Fukuda, Hisanori Matsuura, Ryoji Kiyama

**Affiliations:** 1 Doctoral Program of Neuromotor Science, Graduate School of Health Sciences, Kagoshima University, Kagoshima City, Kagoshima, Japan; 2 Department of Physical Therapy, School of Health Sciences, Faculty of Medicine, Kagoshima University, Kagoshima City, Kagoshima, Japan; 3 Department of Information, Artificial Intelligence and Data Science, Faculty of Engineering, Daiichi Institute of Technology, Kirishima City, Kagoshima, Japan; 4 Department of Rehabilitation, Faculty of Health Sciences, Kumamoto Health Science University, Kumamoto City, Kumamoto, Japan; Carol Davila University of Medicine and Pharmacy: Universitatea de Medicina si Farmacie Carol Davila din Bucuresti, ROMANIA

## Abstract

Rotator cuff tears decrease glenohumeral joint stability; however, surrounding uninjured muscles could compensate for the reduced function of injured muscles to maintain joint stability. This study set out to analyze compensatory mechanisms that could maintain glenohumeral joint stability following rotator cuff tears of varying severity during normal daily life, using a musculoskeletal model simulation of rotator cuff tears. Fifteen healthy males performed 6 activities including shoulder flexion and abduction, contralateral shoulder reaching, head reaching, and lifting a 1 kg object placed on low and high shelves. Glenohumeral joint stability and scapulohumeral muscle forces were estimated using an intact model and 7 models with complex injuries to the supraspinatus, infraspinatus, and subscapularis muscles. Joint stability was quantified using the positional relationship between joint reaction force and the glenoid. Relationships between changes in glenohumeral joint stability and scapulohumeral muscle forces, in the different models, were analyzed. Glenohumeral joint stability decreased in all activities according to rotator cuff tear severity. Decreased glenohumeral joint stability was more pronounced with lower arm elevation angles and greater severity of rotator cuff tear. At this moment, increase in teres minor and long head of the biceps were observed in the rotator cuff tear models compared with the Intact model. Teres minor force increased in the isolated supraspinatus model during all activities, and long head of biceps forces increased in Flexion, Abduction, and Head reaching activities. These increases in muscle forces were significantly correlated with joint instability, indicating their contribution to stabilization of the glenohumeral joint. In conclusion, in rotator cuff tear models of differing severity, increased uninjured muscle forces compensated for decreased glenohumeral joint movement. Compensatory mechanisms differed according to the pattern and severity of rotator cuff tears, as well as activity type.

## Introduction

Rotator cuff tears (RCT) are the most common shoulder disorder and functional impairment due to RCT often negatively affects daily life. The prevalence of RCT is estimated to be 20.7% and increases with age: 25.6% of individuals above 60 y; 45.8% of individuals above 70 y; 50.0% of individuals above 80 y [1]. Rotator cuff muscle dysfunction following severe RCT causes decreased glenohumeral compression force, resulting in joint instability [2,3]. Subsequent dysfunction associated with joint instability can limit activities of daily living (ADL), leading to a decline in independence. In contrast, some individuals with RCT do not experience difficulties with daily activities [1,4,5]. This implies that surrounding, uninjured muscles could compensate for the impaired function of injured muscles to help maintain joint stability [6].

RCT vary in pattern, ranging from isolated partial tear to massive tears that include the supraspinatus (SSP), infraspinatus (ISP), and subscapularis (SSC) [7]. Most RCT involve the SSP, with approximately 35% being isolated SSP tears; the remaining cases often include more complex injuries [8]. Compensatory mechanisms are expected to differ depending on the tear pattern. Therefore, an understanding of the mechanisms adopted for each RCT pattern is useful for planning rehabilitation. However, it is difficult to directly analyze, glenohumeral joint stability and scapulohumeral muscle forces, in vivo, and only a few reports have addressed ADL.

Musculoskeletal model simulation can analyze joint reaction force (JRF) and muscle force during activities and quantify joint stability based on positional relationships between the JRF and glenoid. Several studies have defined shoulder joint instability as a glenohumeral JRF away from the center of the glenoid or outside the edge [2,6,9–11]. Another simulation study indicates that glenohumeral joint stability decreases according to RCT severity during ADL [10]. According to Holscher et al., weakness of the superior rotator cuff, including SSP and ISP, leads to a JRF closer to the upper glenoid rim and is related to joint instability [12]. Furthermore, another study using RCT modeling shows that the teres minor (Tm), which generates inferior force, can compensate for glenohumeral joint stability following RCT severity during arm elevation [6]. However, few studies have investigated the relationship between glenohumeral joint stability and scapulohumeral muscle forces during ADL. Moreover, the compensatory mechanism is unclear.

The purpose of this study was to estimate glenohumeral joint stability and scapulohumeral muscle forces during ADL and to analyze compensatory mechanisms for glenohumeral joint instability according to RCT severity using musculoskeletal model simulation with RCT models. We compared muscle forces estimated from the Intact and RCT models. We hypothesized that uninjured muscle forces increased with RCT severity and contributed to the compensatory glenohumeral joint stability. In addition, contributing muscles differed according to tear pattern and activities.

## Materials and methods

### Participants

Fifteen healthy males, with no history of neurological or upper limb pathology (age, 22.2 ± 1.8 y; height, 171.5 ± 6.3 cm; weight, 63.5 ± 7.1 kg) participated in this study. Participants were recruited between 3 July 2022 and 6 December 2022, and all participants provided signed informed consent. The sample size was determined based on previous studies that analyzed glenohumeral joint stability using musculoskeletal model simulation [6]. This study was approved by the Ethics Committee on Epidemiological and Related Studies at the Sakuragaoka campus of Kagoshima University (approval number: 220022 Epi ver. Revision 1).

### Activities of daily living and motion capture

Participants performed shoulder flexion, abduction (Flexion and Abduction), and 4 ADL including contralateral shoulder reaching (Shoulder), head reaching (Head), and lifting up a 1 kg object under two height conditions (UpL and UpH). These activities were measured using a motion capture system, and glenohumeral joint stability and scapulohumeral muscle forces were estimated with the shoulder model from the AnyBody modeling system (AMMR v.2.4.3, AnyBody7.3, AnyBody Technology, Denmark). Then, comparisons among the Intact model and various RCT models were conducted.

Motion capture was performed using an 8-camera OptiTrack Flex 13 system (NaturalPoint, Corvallis, OR, USA) with reflective markers placed on the body surface with a sampling frequency of 100 Hz. Twenty-four reflective markers were placed according to a plug-in-gait marker set, with an additional rigid cluster of 3 markers on the upper arm and forearm. All participants were right dominant, and activities were analyzed according to the dominant hand.

**Fig 1:**
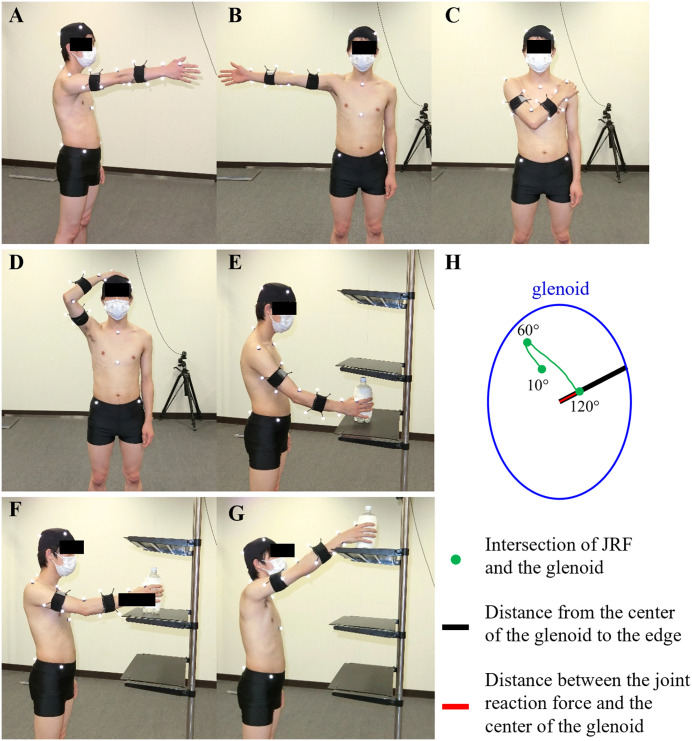
ADL activities. A) Flexion, B) Abduction, C) Shoulder, D) Head, E to F) UpL, F to G) UpH, H) Glenohumeral joint stability.

In Flexion and Abduction, the starting position was static upright with arm drooped and the ending position was full arm elevation (Fig 1A and 1B). Participants were asked to complete elevation in 5 s. We analyzed arm elevation angle up to 120° of Flexion and Abduction for the required range of motion of daily life. Shoulder and Head were performed to mimic axilla washing and hair washing (Fig 1C and 1D). The starting position was static upright with arm drooped and the ending position was when touching the contralateral shoulder or vertex of the head with the right arm. UpL and UpH were performed at the anterior-median line on shelves of 3 heights: superior-anterior iliac spine level, eye level, and an intermediate position between them [13]. In this study, UpL was the lifting activity from the superior-anterior iliac spine level to intermediate position (Fig 1E to 1F) and UpH was from the intermediate position to eye level (Fig 1F to 1G). UpL and UpH were analyzed during movement of a 1 kg object away from the lower shelf and until it was placed on the upper shelf under the participant’s spontaneous velocity. Motion capture data was filtered using a Butterworth low-pass filter at a 6 Hz cut-off frequency for further analysis.

### Musculoskeletal model simulation

The musculoskeletal model simulation software and the shoulder arm model in AnyBody Managed Model Repository were used to estimate glenohumeral joint stability and scapulohumeral muscle forces using inverse dynamics and optimization [14,15]. This shoulder model was originally developed by the Dutch Shoulder Group [16] and incorporates detailed bone geometry of the ribcage, clavicle, scapula, and humerus, together with 118 muscle-tendon units and 5 joints, including the acromioclavicular, sternoclavicular, glenohumeral, elbow, and wrist joints. This model has been validated as a physiologically accurate musculoskeletal model by comparing simulated JRF with those measured in vivo and muscle activity measured using surface electromyography [17,18]; it has been widely used to analyze the kinematics and kinetics of joints and muscles in simulated shoulder problems [12,17]. In this study, we used the default model (Intact model) and RCT models with simulated RCT.

Each muscle used a 3-element muscle model, consisting of a Hill-type contractile element, a parallel-elastic element, and a series-elastic element. Optimization was performed to minimize the sum of the cubes of the muscle load expressed by the ratio of exerted muscle output to maximum muscle strength of each muscle. Furthermore, stabilizing function was modeled by constraining the glenohumeral JRF to pass through the glenoid [19]. If the JRF passed outside the glenoid, the simulation was aborted. The glenoid was defined as an ellipse in anterior-posterior (28 mm) and superior-inferior (38 mm) directions [20]. Joint stability was quantified from a positional relationship between the JRF and glenoid. The following formula was used:


$$Joint\;stability=\text{1}-\frac{distance\;between\;JRF\;intersection\;and\;center\;of\;glenoid\;}{distance\;from\;center\;of\;glenoid\;to\;the\;edge}$$

where, JRF intersection was the intersection of the JRF and glenoid defined as an ellipse. The closer the calculated value was to 0, the closer the glenohumeral JRF passed to the edge of the glenoid, indicating joint instability (Fig 1H). In a typical pattern, the intersection of JRF moved superiorly and posteriorly from a neutral position, decreasing joint stability, and then moved inferiorly, increasing joint stability, according to arm elevation, consistent with a previous study [6,10,11].

Muscle forces were analyzed for the following 8 scapulohumeral muscles: SSP, ISP, SSC, Tm, anterior, middle and posterior deltoid (AD, MD, and PD) and long head of biceps (LHB). The validity of muscle forces estimated by musculoskeletal model simulation has been verified in previous studies [15,16].

### Rotator cuff tear model

We developed 7 tear models that simulated RCT, in addition to the Intact model. RCT models were simulated by reduced maximum muscle strength of the SSP, ISP, and SSC based on previous simulation and epidemiological studies that investigated the prevalence of tear patterns [8,21]. For RCT severity ranging from no RCT to massive, three-muscles tears were modeled, as described in Table 1. In RCT models, the strength of each injured muscle declined to 75%, 50%, 25%, and 0%, respectively. We simulated isolated SSP tear (S50, S0), SSP and ISP tears (S0/IS75, S0/IS50, S0/IS25), and SSP, ISP, and SSC tears (S0/IS25/SS50).

**Table 1:** Models of rotator cuff tear (RCT) severity were simulated to reduce maximum muscle strength.

Tear Severity	Supraspinatus (S)	Infraspinatus (IS)	Subscapularis (SS)	Teres Minor (TM)
Intact	100%	100%	100%	100%
S50	50%	100%	100%	100%
S0	0%	100%	100%	100%
S0/SS50	0%	100%	50%	100%
S0/IS75	0%	75%	100%	100%
S0/IS50	0%	50%	100%	100%
S0/IS25	0%	25%	100%	100%
S0/IS25/SS50	0%	25%	50%	100%

Based on previous studies, we focused our modeling on the tear patterns most commonly seen in clinical practice. Percentages of the Intact model’s muscle strength were 100% in all muscles. Tear muscle strength ranged from partial (75%, 50%, and 25%) to full (0%). 75% muscle strength indicated a small tear, 50% indicated a medium tear, and 25% indicated a large tear. Intact: Full muscle strength in all muscles. S50: Isolated partial supraspinatus tear. S0: Isolated full supraspinatus tear. S0/SS50: Full supraspinatus tear; partial subscapularis tear. S0/IS75: Full supraspinatus tear; partial infraspinatus tear. S0/IS50: Full supraspinatus tear; partial infraspinatus tear. S0/IS25: Full supraspinatus tear; partial infraspinatus tear. S0/IS25/SS50: Full supraspinatus tear, partial infraspinatus tear, and partial subscapularis tear.

### Data and statistical analyses

Arm elevation angle was calculated as the angle between a vertical line and humerus long axis, and kinetics were analyzed every 10° during each activity. The analyzed range of arm elevation angle was determined to be extracted from all participants for each activity. Analyses ranged from 10° to 120° in Flexion and Abduction, from 10° to 40° in Shoulder, from 10° to 110° in Head, from 40° to 60° in UpL, and from 80° to 100° in UpH.

Glenohumeral joint stability and scapulohumeral muscle forces estimated from the Intact model and RCT models were compared every 10° of arm elevation to analyze the dynamic stabilization mechanism [22]. The increased muscle forces in RCT models compared with the Intact model was designed to contribute toward compensation for lack of joint stability or lack of moment [6,11]. JRF was estimated according to the coordinate system recommended by the International Society of Biomechanics [23]. Muscle forces were normalized to each participant’s body weight. The means of 5 trials in each activity were analyzed, and data were processed using Matlab (2020a, Mathworks). Normality of distribution was tested using the Shapiro-Wilk test. If normality of distribution could be assumed, data were analyzed using one-way repeated measures analysis of variance (ANOVA) followed by paired t-tests. If the normality of distribution could not be assumed, data were analyzed using the Friedman test followed by the Wilcoxon signed-rank test. Both post hoc tests were performed to compare the Intact model and RCT models, and they were adjusted using the Bonferroni correction. Effect sizes for ANOVA and Friedman test were also estimated using eta squared and Kendall’s W.

Additionally, correlation analysis was performed to examine the relationship between changes in muscle forces and glenohumeral joint stability for muscles expected to produce a downward force on the humeral head, including the infraspinatus, subscapularis, teres minor, and long head of the biceps [2,6,9,24,25]. Pearson’s or Spearman’s correlation coefficient were used depending on the results of the Shapiro-Wilk test. A negative correlation, indicating an increase in muscle forces when joint stability decreased, was considered an indicator of a compensatory contribution to glenohumeral joint stability. All statistical tests were performed using Matlab Statistics and Machine Learning Toolbox (2020a, Mathworks) and the significance level was set to P < 0.05. Results are presented as mean and standard deviations.

## Results

**Fig 2:**
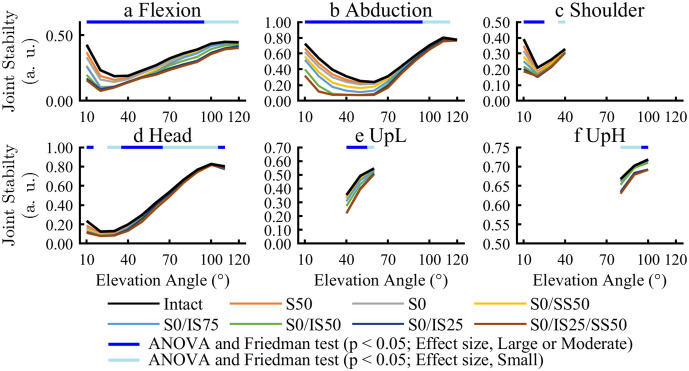
Comparison of joint stability. Joint stability ranged from 0 to 1, with a lower value indicating instability. The upper bar indicates the results of ANOVA and Friedman test.

Simulation of all activities was completed for all RCT models. Glenohumeral joint stability significantly decreased for all RCT models according to RCT severity in all activities except for UpH for the isolated SSP RCT model (S0, S50; Fig 2; S1 Fig). In all activities, glenohumeral joint stability showed a low value in arm elevation at a low angle.

**Fig 3:**
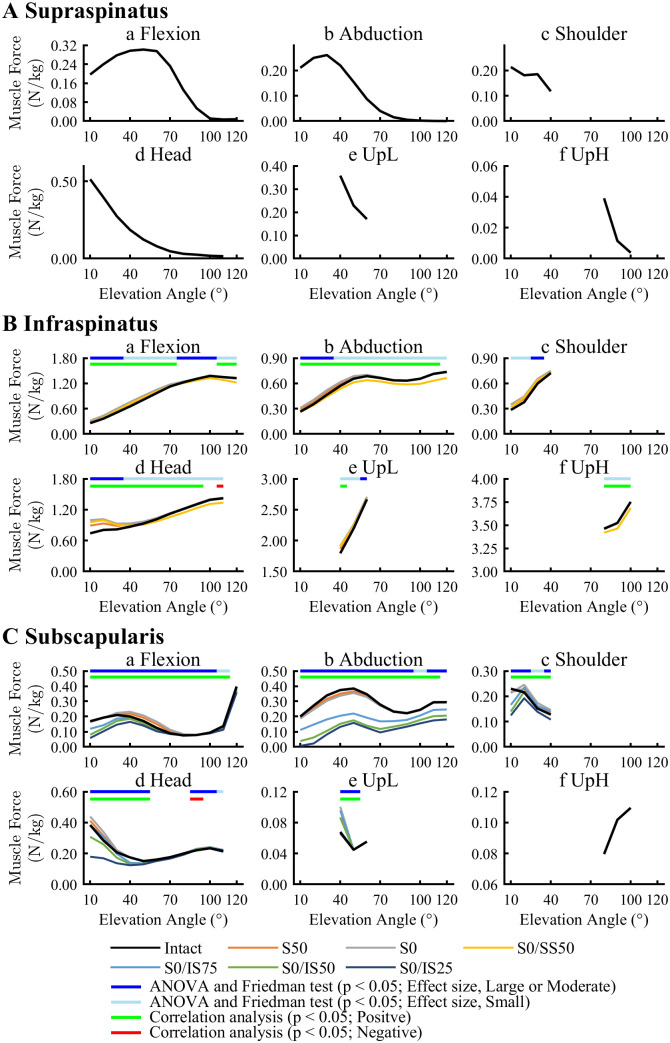
Muscle forces of the supraspinatus (A), infraspinatus (B), and subscapularis (C). Muscle forces were normalized by body weight (N/kg). The supraspinatus, infraspinatus, and subscapularis forces are only shown in the models without their own tears. The upper bar indicates the results of ANOVA and Friedman test, as well as correlation analysis of the relationship between changes in muscle forces and glenohumeral joint stability.

SSP forces during Flexion and Abduction activities showed a peak between 40° to 60° on 20° to 40° of arm elevation for the Intact model, respectively. In the other activities, SSP forces were maximum at the starting position (Fig 3A). The SSP generated forces mainly within the joint angle corresponding to the phase of decreasing glenohumeral joint stability in cuff tear models. In contrast, ISP forces for the Intact model were maximum near the final position in all activities. An isolated SSP tear (S50, S0) increased ISP forces in the early phases across all activities except for UpH, compared with the Intact model (Fig 3B; S1 Fig). Also, isolated SSP tear (S50, S0) significantly increased SSC forces on arm elevation of 40° to 60° during Flexion, and at 20° during Shoulder activities (Fig 3C; S1 Fig). Inversely, SSC forces during Abduction in all RCT models, as well as during Flexion and Head activities in ISP tear models, was less than that in the Intact model. In correlation analysis, ISP and SSC forces showed significant positive correlations with joint stability, except for a specific range.

**Fig 4:**
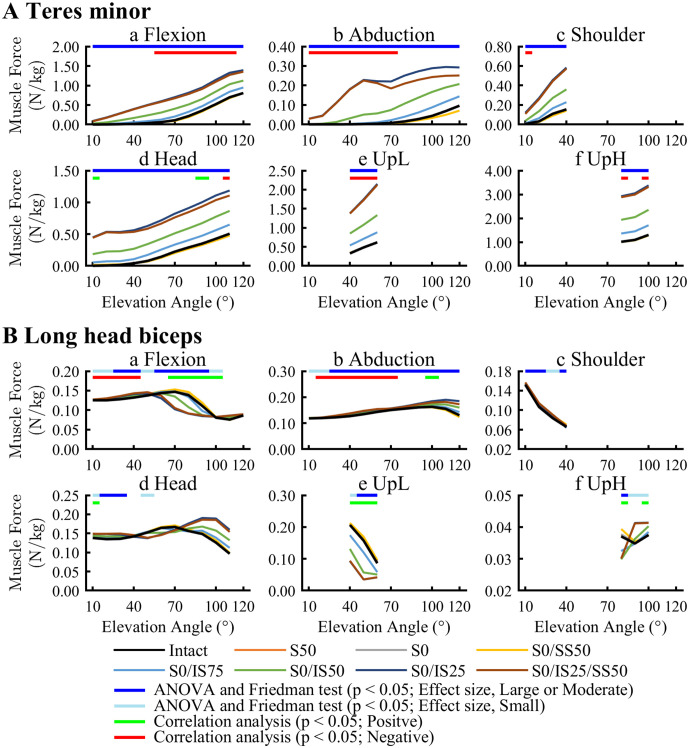
Muscle forces of teres minor (A) and long head biceps (B). Muscle forces were normalized by body weight (N/kg). The upper bar indicates the results of ANOVA and Friedman test, as well as correlation analysis of the relationship between changes in muscle forces and glenohumeral joint stability.

Tm forces significantly increased for RCT models including an ISP tear (S0/IS75, S0/IS50, S0/IS25, S0/IS25/SS50) compared with the Intact model in all activities (Fig 4A; S2 Fig). Increase in Tm forces were strongly dependent on RCT severity; however, Tm forces also showed a significant increase in some cases for RCT models without an ISP tear (S50, S0, and S0/SS50), these increases were negligible. In correlation analysis, Tm forces showed broader associations with significant negative correlations across multiple activities, including 60° to 110° of arm elevation in Flexion, 10° to 70° in Abduction, 40° to 60° in UpL, and 80° and 100° in UpH.

LHB forces increased significantly for RCT models during all activities except Shoulder and lifting activities, compared with the Intact model (Fig 4B; S2 Fig). Characteristic patterns emerged according to RCT severity and activity type. LHB forces increased throughout almost the entire motion during the Abduction activity for the severe RCT models (S0/IS75, S0/IS50, S0/IS25, S0/IS25/SS50). In Flexion and Head, LHB forces significantly increased in the early phase of arm elevation for all RCT models. In correlation analysis, LHB forces showed a significant negative correlation at 10° to 40° in Flexion and 20° to 70° in Abduction.

**Fig 5:**
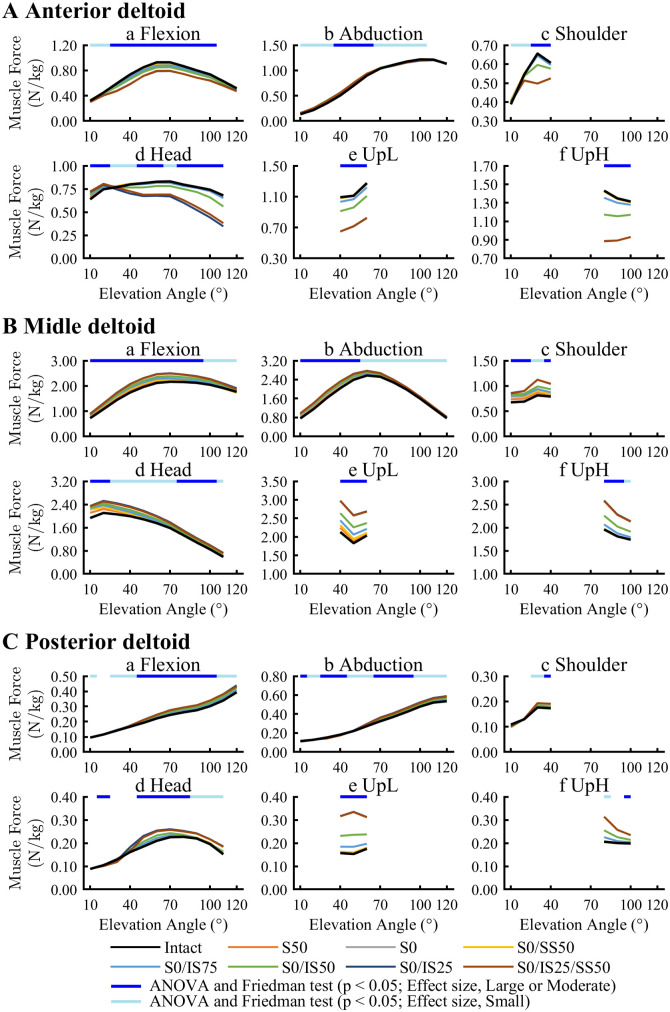
Muscle forces of anterior (A), middle (B), posterior (C) deltoids. Muscle forces were normalized by body weight (N/kg). Muscle forces were normalized by body weight (N/kg). The upper bar indicates the results of ANOVA and Friedman test.

AD forces significantly increased for RCT models compared with the Intact model at 10° to 50° during Abduction, and at 10° to 30° during Head (Fig 5A; S3 Fig). However, AD forces in Flexion, Shoulder and lifting activities significantly decreased for some RCT models. MD forces significantly increased for all RCT models following RCT severity in all activities (Fig 5B; S3 Fig). MD forces increased throughout the entire motion in all activities and was greater in a wide range of arm elevation compared to AD forces. PD forces significantly increased in all activities except UpH according to RCT severity (Fig 5C: S3 Fig).

## Discussion

In this study, we analyzed glenohumeral joint stability and uninjured muscle forces following different severities of RCT; we focused on ADL using musculoskeletal model simulation. The results suggested that glenohumeral joint stability decreased in all activities, but increasing uninjured muscle forces including Tm and LHB improved stability. Muscles involved in compensation differed depending on the activities, limb position, and severity of RCT in the models.

In all activities, glenohumeral joint stability significantly decreased compared with the Intact model, particularly at low arm position and according to the severity of the RCT. The joint angle where joint stability decreased coincided with the angle at which the SSP muscle was active, suggesting that SSP tears further decreased stability. The joint angle at which joint stability decreased was characterized by increases in various muscle forces. In this study, we hypothesized that, when glenohumeral joint stability was decreased, increased forces from the surrounding muscles helped compensate for this weakness.

Correlation analysis revealed negative associations between Tm and LHB forces and glenohumeral joint stability, indicating compensatory increases in these muscle forces in response to decreased stability in line with RCT severity. Tm forces significantly increased for RCT models including ISP tear (S0/IS75, S0/IS50, S0/IS25, S0/IS25/SS50) in all activities and as RCT severity increased [6]**.** Although these enhanced forces were not found in the isolated SSP tear model, this trend was consistent with prior literature [6]. Tm appeared hypertrophic for RCT involving an ISP tear [26,27] and we proposed that this hypertrophy helped to compensate for ISP dysfunction. A previous study reported that Tm compensated for ISP in a particular arm elevation between mid and late phase for RCT involving an ISP tear [24]. In the present study, these increases contributed to joint stability within specific ranges of arm elevation: late phase during Flexion and early to mid-phase during Abduction. Similarly, compensatory increases in Tm forces were also observed during the lifting activity. Previous studies report that shoulder adductor muscles, such as Tm, generate the greatest torque when external loads are applied, and this mechanism may explain the increased Tm forces observed during lifting activities [28]. These increased Tm forces compensated for both decreased joint stability and joint moment depending on the upper arm limb position and RCT severity. However, during ADL (activities such as Shoulder and Head) the compensatory mechanisms of the Tm for glenohumeral joint stability appeared to be limited.

Increase in LHB forces showed a negative correlation with decreased joint stability in the early phase of Flexion and Abduction. LHB contributed to glenohumeral joint stability by compressing the humeral head to the glenoid and reducing shear force [25]. Randomized controlled trials report the concomitant occurrence of inflammation and rupture of the LHB tendon, possibly resulting from increased compensatory loading and potentially contributing to secondary disorder. Otherwise, during ADL, LHB forces did not appear to compensate for glenohumeral joint stability. We considered that LHB was involved in elbow joint motion as well as the shoulder joint and was strongly influenced by movement and limb position. Previous EMG studies suggest that the contribution of LHB activation decreases in internally rotated shoulder positions compared with neutral or externally rotated positions [29]. As several ADL tasks in this study involved internal rotation, this likely explains the observed lack of compensation. These findings suggest that an appropriate compensatory pattern takes place according to tear pattern and limb position, in cases of RCT.

Increase in ISP and SSP showed a positive correlation with an increase in joint stability. These muscles primarily contribute to compensating for the generation of joint moment, rather than maintaining joint stability. The deltoids, which generate superior forces on the humerus, would have a similar role to those muscles. Increased ISP forces occurred in the isolated SSP tear models at the early phase during all activities except for UpH activity. A previous study revealed running of the ISP muscle is similar to the SSP and increased ISP force during abduction compensates for loss of SSP function [24]. Our results suggested similar trends for other ADL activities. SSC forces also significantly increased for isolated SSP tear models in Flexion, Head, and Shoulder on partial range of arm elevation.

AD forces significantly increased throughout the early to mid-phase of Abduction and early phase of the Head activity although the forces decreased in Flexion, Shoulder and lifting activities. Meanwhile, MD and PD forces significantly increased in all activities, except for UpH in PD. Greater deltoid force was required to compensate for lack of shoulder elevation moment following declined SSP function during early phase [24,30]. However, the deltoid also generated superior shear force and translation of the humeral head through elevation torque at an early phase [31]. An increased superior shear force caused an increased load on the labrum or impingement [32]. Therefore, increased forces to compensate for elevation moment may influence a secondary dysfunction of glenohumeral joint stability. Consequently, to compensate for impaired shoulder joint function with the deltoid, coordination with the TM and LHB is necessary.

Although PD forces in the RCT model increased in all activities except for UpH, decreased AD forces were found in all activities except for Abduction. Other studies have shown a compensatory increase in posterior muscles of the shoulder, including Tm, MD, and PD, following ISP tear [21]. Increased PD forces and decreased AD forces might contribute to joint stabilization by controlling the vector of forces in an anterior-posterior direction. Similarly, SSC forces decreased in Flexion, Shoulder, and Head activities in the ISP tear model, and in Abduction activities in all levels of RCT severity. Ackland et al. [24] recommended that loss of ISP function resulted in a muscle recruitment pattern that preferentially deactivated SSC in order to maintain a muscle torque-balance in the transverse plane [11]. Thus, our results could have resulted in a similar phenomenon.

In preset study, glenohumeral joint stability decreased for all activities following reduced maximum SSP, ISP, and SSC forces. The compensating muscles differed according to activity, RCT severity, and arm position. Meanwhile, previous cadaver studies, analyzed glenohumeral joint stability by changing the movement of these muscles according to limb position [24,33]. We suggest that glenohumeral joint stability is compensated for by adjacent uninjured muscles and that clinicians need to pay attention to these mechanisms. Notably, Tm and LHB appear to play key roles in directly compensating for decreased glenohumeral joint stability, whereas ISP and deltoid may act primarily to supplement the loss of joint moment. Although previous studies have addressed glenohumeral joint stability and muscle compensation—focusing on ADL and arm elevation activities separately, the present study is, to our knowledge, the first to comprehensively analyze both joint stability and compensatory muscle forces during ADL [10,11]. The present results suggest that, in individuals with RCT, targeted exercises for the Tm and LHB may facilitate the development of compensatory movement patterns. Rehabilitation programs should therefore combine strength training for these muscles with task-specific interventions that promote appropriate compensatory strategies.

There were 3 limitations to this study. First, participants were healthy young males, which limits the generalizability of the findings to older populations who are more commonly affected by RCT. Older individuals have 16.5% less muscle mass in the upper extremity as a whole than younger persons [34], which may influence muscle recruitment patterns. Therefore, the compensatory strategies observed in this study may differ from those in older or symptomatic populations. Second, we did not consider scapulothoracic motion in the patients with RCT and changes affected glenohumeral JRF [35]. In addition, scapulohumeral rhythm is smaller in individuals with RCT than in healthy people [36]; this alteration would be expected to change muscle forces, and the compensatory mechanism would also be different. Therefore, the effect of altered scapulothoracic motion on glenohumeral joint stability remains unclear and warrants further investigation. Future studies may help clarify this relationship and provide data relevant to clinical management. Finally, in this study, we simulated RCT by reducing maximum muscle strength, but the excessive co-contraction of the shoulder periarticular muscles and pain observed in patients with RCT were not analyzed. Further studies that take these findings into account are needed.

## Conclusions

This study showed that decreased glenohumeral joint stability during ADL, dependent on RCT severity, was partially protected by a compensatory mechanism involving nearby uninjured muscles. The muscles involved in the compensatory mechanism were shown to be different depending on RCT pattern and severity, type of activity, and limb position. Additionally, the altered scapulohumeral rhythm observed in patients with RCT may influence the force of uninjured muscles and the stability of the glenohumeral joint, which should be explored in future research.

## Supporting information

S1 Fig Differences in glenohumeral joint stability (A) and muscle force of the infraspinatus (B), and subscapularis (C) in models with and without cuff tears. The black bar indicates a significant increase compared with the Intact model, and the gray bar indicates a significant decrease compared with the Intact model.
(TIF)

S2 Fig Differences in the muscle force of teres minor (A) and long head biceps (B) in models with and without cuff tears. The black bar indicates a significant increase in muscle force compared with the Intact model, and the gray bar indicates a significant decrease in muscle force compared with the Intact model.
(TIF)

S3 Fig Differences in the muscle force of anterior (A), middle (B), and posterior (C) deltoids in models with and without cuff tears. The black bar indicates a significant increase in muscle force compared with the Intact model, and the gray bar indicates a significant decrease in muscle force compared with the Intact model.
(TIF)
