# Supplementary figures and images for: Compensatory mechanisms to maintain glenohumeral joint stability in rotator cuff tears of differing severity during activities of daily living: A musculoskeletal model simulation study
Source: PLoS One. 2025 Oct 31;20(10):e0335647. doi: 10.1371/journal.pone.0335647 (PMC12578153; doi:10.1371/journal.pone.0335647)

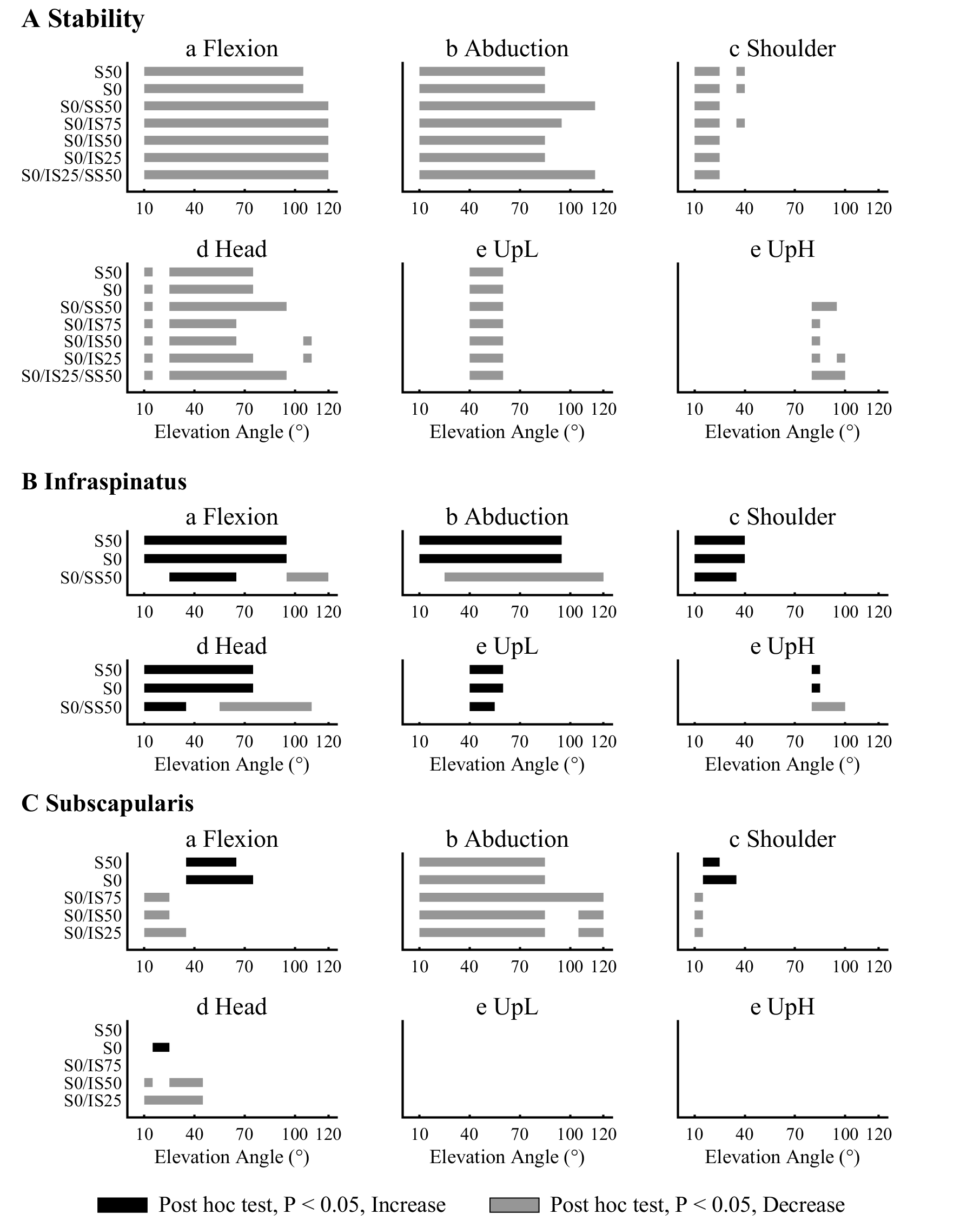

Supplement: S1 Fig — The black bar indicates a significant increase compared with the Intact model, and the gray bar indicates a significant decrease compared with the Intact model. (TIF) [file pone.0335647.s001.tif]

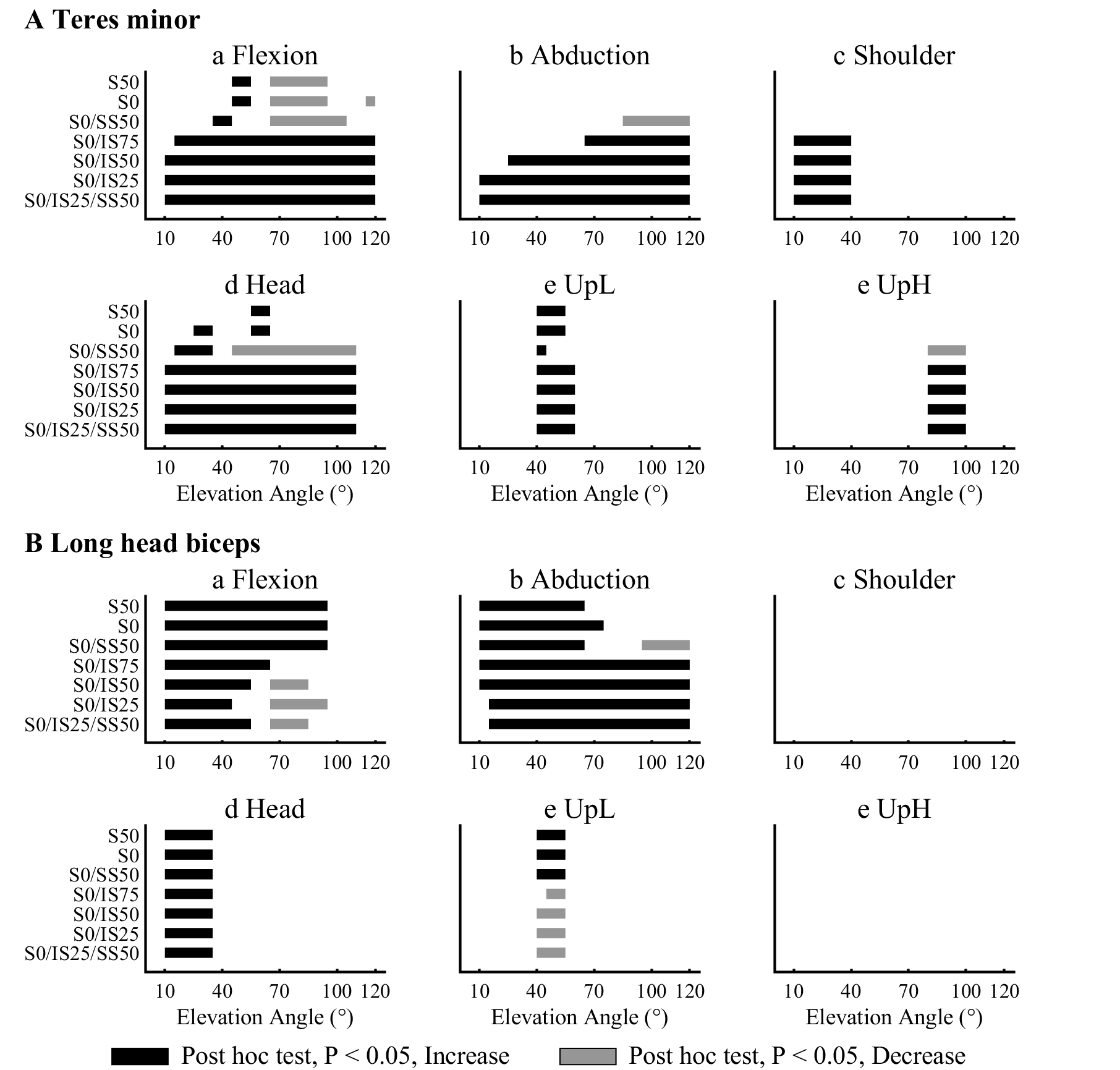

Supplement: S2 Fig — The black bar indicates a significant increase in muscle force compared with the Intact model, and the gray bar indicates a significant decrease in muscle force compared with the Intact model. (TIF) [file pone.0335647.s002.tif]

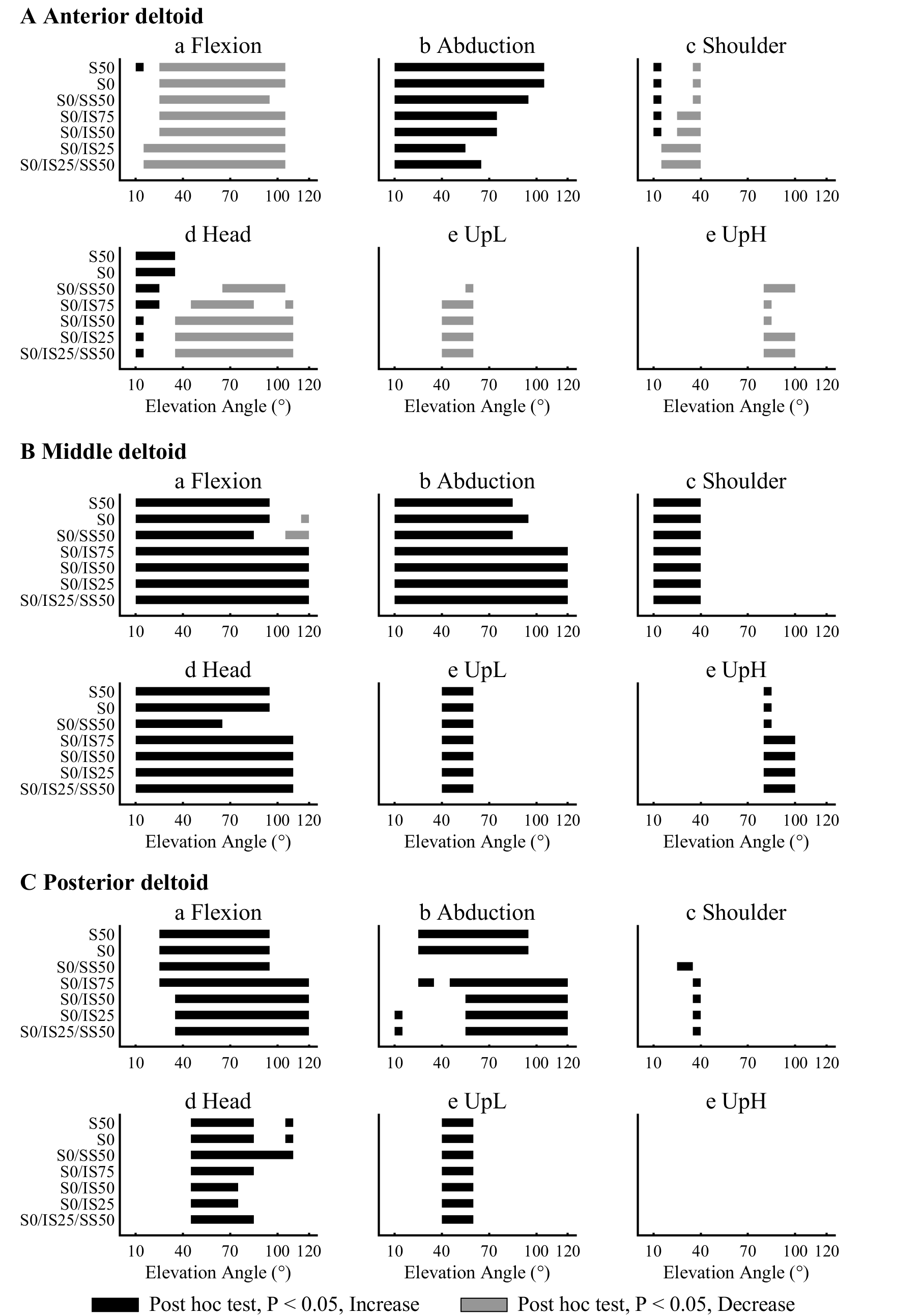

Supplement: S3 Fig — The black bar indicates a significant increase in muscle force compared with the Intact model, and the gray bar indicates a significant decrease in muscle force compared with the Intact model. (TIF) [file pone.0335647.s003.tif]
